# Microbial Inventory of Deeply Buried Oceanic Crust from a Young Ridge Flank

**DOI:** 10.3389/fmicb.2016.00820

**Published:** 2016-05-27

**Authors:** Steffen L. Jørgensen, Rui Zhao

**Affiliations:** Department of Biology, Centre for Geobiology, University of BergenBergen, Norway

**Keywords:** deep biosphere, oceanic crust, geobiology, cell abundance, community structure, endolitihic community

## Abstract

The deep marine biosphere has over the past decades been exposed as an immense habitat for microorganisms with wide-reaching implications for our understanding of life on Earth. Recent advances in knowledge concerning this biosphere have been achieved mainly through extensive microbial and geochemical studies of deep marine sediments. However, the oceanic crust buried beneath the sediments, is still largely unexplored with respect to even the most fundamental questions related to microbial life. Here, we present quantitative and qualitative data related to the microbial inventory from 33 deeply buried basaltic rocks collected at two different locations, penetrating 300 vertical meters into the upper oceanic crust on the west flank of the Mid-Atlantic spreading ridge. We use quantitative PCR and sequencing of 16S rRNA gene amplicons to estimate cell abundances and to profile the community structure. Our data suggest that the number of cells is relatively stable at ~10^4^ per gram of rock irrespectively of sampling site and depth. Further, we show that *Proteobacteria*, especially *Gammaproteobacteria* dominate the microbial assemblage across all investigated samples, with Archaea, in general, represented by < 1% of the community. In addition, we show that the communities within the crust are distinct from the overlying sediment. However, many of their respective microbial inhabitants are shared between the two biomes, but with markedly different relative distributions. Our study provides fundamental information with respect to abundance, distribution, and identity of microorganisms in the upper oceanic crust.

## Introduction

Every day ~100 billion cubic meters of bottom seawater are transported down into the permeable upper oceanic crust. Within this gigantic aquifer system oxic seawater circulates and reacts with reduced igneous rocks before eventually recharging back into the oceans 10^3^–10^4^ years later (Wheat et al., 2003; Orcutt et al., 2011). Consequently, the chemical composition of fluids and rocks are strongly altered, with wide-reaching ramifications throughout the marine system (Fisher and Becker, 2000; Bach and Edwards, 2003; Bach et al., 2004). Strong evidence exist for an abundant microbial community residing within this subsurface crustal basaltic aquifer (Giovannoni et al., 1996; Torsvik et al., 1998; Fisk et al., 2003; Lysnes et al., 2004; Orcutt et al., 2011; Nigro et al., 2012; Lever et al., 2013) where microbial activity is believed to influence basalt alteration and mineral dissolution rates (Thorseth et al., 1995; Fisk et al., 1998; Furnes et al., 2001b; Storrie-Lombardi and Fisk, 2004; Kruber et al., 2008).

Endolithic microorganisms in subsurface basalt were first reported two decades ago from a drilling expedition to the Costa Rica Rift zone (Ocean Drilling Program Leg, ODP Leg 148). The presence and activity of microorganisms were inferred via detection of biosignatures including: (i) microscopic tubular structures in which DNA could be detected by staining (Thorseth et al., 1995; Giovannoni et al., 1996), (ii) targeting and localization of intact and active cells via *in situ* fluorescent hybridization (FISH) (Torsvik et al., 1998), and (iii) site-specific nitrogen and carbon enrichment in the altered tubular structures (Giovannoni et al., 1996; Torsvik et al., 1998). These results were later supported by drilling in the Australian Antarctic Discordance (ODP Leg 187) where, in addition to corroborating textural, geochemical, and molecular observations (Furnes et al., 2001a,b; Thorseth et al., 2003), microbial DNA (16S rRNA genes) from subsurface samples was for the first time successfully amplified and sequenced (Lysnes et al., 2004). Despite limited sequencing depth, this analysis revealed a unique microbial population dominated by the bacterial phyla *Gammaproteobacteria, Actinobacteria, Bacteroidetes, Chloroflexi*, and *Firmicutes*, and different from those in the above sediment and seawater.

Besides the pioneering work outlined above only a few additional microbial studies have directly investigated native subsurface igneous rocks (Fisk et al., 2003; Mason et al., 2010; Lever et al., 2013; Orcutt et al., 2015). Thus, this habitat is heavily under-studied, a fact that can be largely attributed to the immense technical and economic challenges involved in the sampling of deeply buried oceanic crust. Consequently, most of our knowledge about the crustal biosphere originates from samples exposed at the seafloor (Orcutt et al., 2011; Edwards et al., 2012b; Orcutt and Edwards, 2014). Seafloor-derived samples, however, are not representative of the subseafloor crustal environment and constitute only a small fraction of the 10^9^ km^3^ of the upper oceanic crust that has been suggested to be habitable (Heberling et al., 2010). In an effort to address these concerns, *in situ* subseafloor observatories, installed primarily at Juan de Fuca Ridge (JdFR), have expanded our understanding of subseafloor water–rock–microbe interactions in a more representative setting (Orcutt et al., 2011). Despite such technological advancement, a number of basic questions cannot easily be inferred from subseafloor observatories, including cell abundances and community structure in native material.

The first dedicated microbial investigation of a low-temperature young ridge flank system was undertaken by the International Ocean Drilling Program (IODP) expedition 336 to North Pond in the North Atlantic gyre (Expedition 336 Scientists, 2012e). The basement in North Pond is covered by a sediment layer (up to 300 m) and is characterized by vigorous crustal fluid circulation driven mainly by advection (Edwards et al., 2012a). The fast fluid circulation results in relatively low fluid temperatures (10–15°C) and seawater-like fluid chemistry, such as high dissolved oxygen concentration at discharge zones [55–191 μM] (Orcutt et al., 2013). Further, a recent study revealed an active and distinct bacterial community in the crustal fluids underneath North Pond (Meyer et al., 2016).

In the present study, we analyse the abundance and structure of microbial communities in deeply buried basaltic rocks in a total of 33 different samples retrieved from the basement underneath North Pond and compare these to the communities in the above sediments. We analyse the samples by means of 16S rRNA gene amplicon libraries and quantitative PCR (qPCR). Our results are among the first to quantify microbial abundances in native subsurface basalt, thus guiding biomass constrains for this globally significant system. Furthermore, our data elucidate the taxonomic identity of native microbial inhabitants suggesting a community capable of facilitating a diverse range of redox reactions. Lastly, we provide evidence that the dispersal scenarios of the sedimentary and crustal microbial inhabitants are intertwined and potentially closely linked.

## Materials and methods

### Sample location, collection, and description

**Figure 1:**
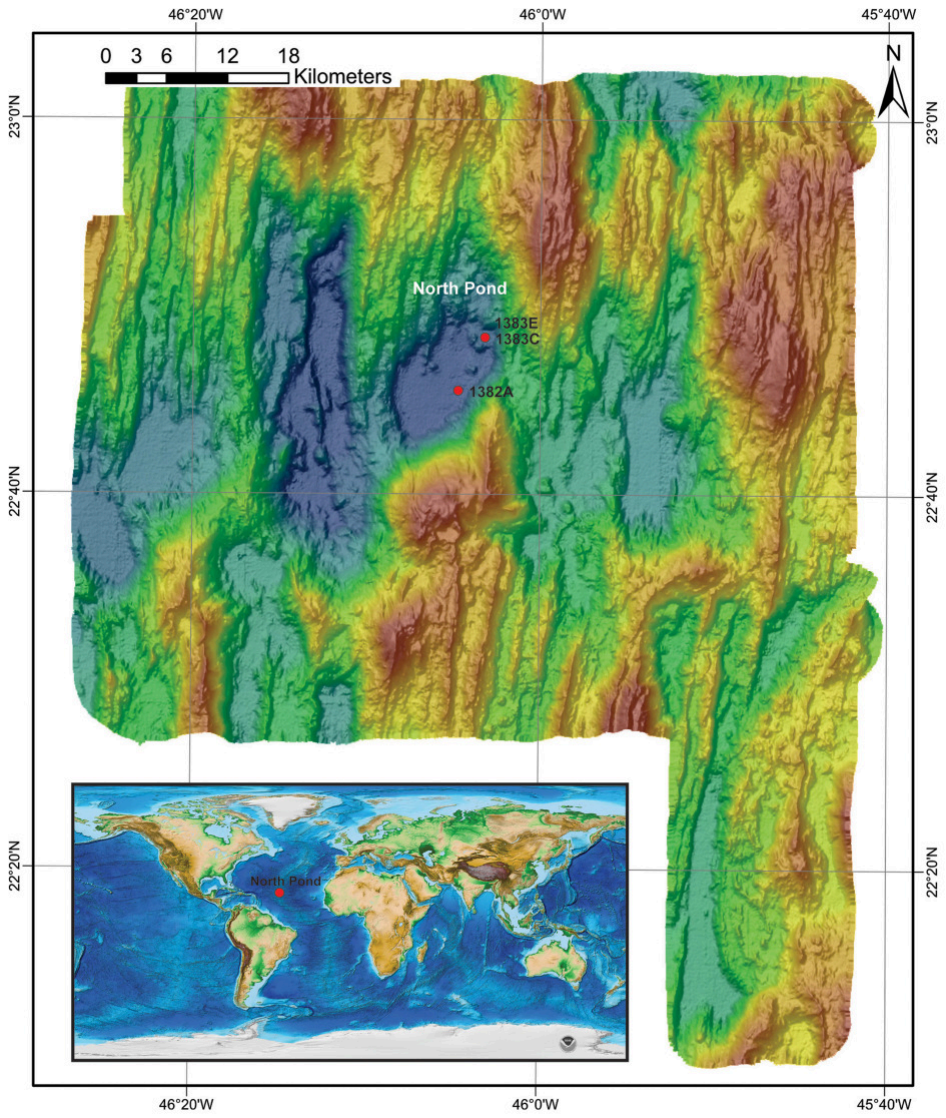
**Bathymetric map with the location of the investigated sites**. Red circles indicate the geographic location of the investigated sites. Insert: global map indicating the position of North Pond in the Mid-Atlantic Ocean (Source: National Centers for environmental Information - NCEI). Modified from Bach (2012).

We investigated a total of 33 subsurface samples (27 from igneous crust and 6 from a sedimentary breccia) collected from North Pond on the west flank of the Mid-Atlantic Ridge. Samples originate from Holes 1382A (22°45.353′N, 46°04.891′W) and 1383C (22°48.1241′N, 46°03.1662′W; Figure 1), both were retrieved using rotary core barrel (RCB) coring. Florescent microspheres were added to the drilling fluid in order to assess potential contamination as described in details elsewhere (Expedition 336 Scientists, 2012d). In order to remove potential contamination introduced during drilling operation, all samples were washed three times in sterile seawater on-board before being sub-sampled into smaller pieces using a chisel and hammer under sterile conditions, as described in details elsewhere (Expedition 336 Scientists, 2012c). The presence of fluorescent microspheres in the wash solution was investigated by microscopy after the last wash. Subsamples (~2 cm^3^) were placed in sterile Whirlpak bags and immediately frozen at −80°C before further processing at the home institute.

Samples follow a depth gradient ranging from ~110 to 200 meter below seafloor (mbsf) in Hole 1382A (16 samples) and 70 to 300 mbsf in Hole 1383C (17 samples). The igneous crust at the two sites likely originates from different volcanic centers fed by a mantle source of variable composition (Expedition 336 Scientists, 2012c). A short overview of sample depths and lithological characteristics can be found in Table 1. For a comprehensive description the reader is recommended to consult the IODP proceedings volume 336 (http://publications.iodp.org/proceedings/336/336title.htm). Following is a brief characterization of each site.

**Table 1:** **General sample description including depth, lithological unit, onboard sample description, and in which group in the hierarchical cluster analysis the microbial community is located**.

**Sample**	**Depth (mbsf)**	**Unit**	**Specific sample description**	**Cluster**
**1382A**				
2R_1C	110	I	Massive, minor red-yellow-brown alteration	4
3R_2B	115	I	Massive, yellow-white-brown alteration in vein	4
3R_3A	117	I	Massive, patchy orange-brown alteration	4
3R_4B	117	I	Massive, mostly brown oxidized halo	4
4R_1B	123	II	Aphyric cryptocrystalline basalt, gray-brown alteration	4
5R_1B	133	II	Massive, aphyric, red alteration	4
6R_1A	142	II	Aphyric, cryptocrystalline, less vesicular, patchy alteration	4
7R_2B	153	III	Aphyric, glassy margin, red, and orange-brown alteration	4
8R_1A	161	IV	Ultramafic, pyroxene, evidence of low-temperature alteration	1
8R_1B	161	IV	Porphyritic basalt	4
8R_2F	162	IV	Orange-brown sediment with small (< 1 mm) basalt clasts	2
8R_3G	163	IV	Gray-brown + orange-brown sediment with small (< 1 mm) basalt clasts	1
8R_4D	163	IV	Sedimentary breccia with basalt clasts, rusty colored, extensive carbon	2
9R_1C	172	IV	Sediment near serpentinized breccia	3
10R_3D	183	V	Medium-grained basalt, massive, porphyritic, pervasive alteration	4
12R_1A	199	VI	Porphyritic basalt, minor alteration	4
**1383C**				
2R_2E	72	I	Aphyric basalt, highly altered, vein with red alteration, light brown alteration	5
3R_1B	77	I	Aphyric basalt, slight alteration, tan alteration deposits	4
4R_1B	87	I	Light tan micrite breccia with altered glass clasts	4
5R_1B_I	97	I	Aphyric basalt, moderately altered, vein with red alteration	5
5R_1B_II	97	I	Aphyric basalt, moderately altered	5
6R_1A	105	I	Aphyric basalt, slight alteration, altered chilled margin, ochre alteration	6
10R_1A	144	II	Light tan micrite with large clasts of altered glass	5
10R_1D	145	II	Phyric basalt, moderately altered, multiple veins, slight red alteration	5
11R_1C	154	II	Phyric basalt, extensive alteration, orange, and olive alteration	5
19R_1B	212	III	Aphyric basalt, highly oxidized, light brown alteration, relatively brittle	5
19R_1A	212	III	Two small pieces, mostly glass, rust alteration	5
20R_1A	219	III	All basalt glass, rust alteration	5
24R_1B	257	III	Aphyric basalt, oxidized, dark orange-brown alteration, fractured	5
24R_1A	256	III	Aphyric basalt glass with rust alteration, vesicles	5
27R_1A	285	III	Aphyric massive basalt, alteration	4
29R_1A	300	III	Aphyric basalt, highly oxidized, thin carbonate veins	6
30R_1A	304	III	Aphyric basalt, oxidized, some fractures, dark orange alteration	5

*Cluster number corresponds to numbers in Figure 4. The sample description presented here is modified from IODP 336 site summaries (Expedition 336 Scientists, 2012a,b)*.

In Hole 1382A basement was located 90 mbsf, however the interval from 93 to 99 mbsf are inferred to be sedimentary (Expedition 336 Scientists, 2012e). A total of 32 meter of upper crustal material was recovered between 110 and 210 mbsf (recovery 32%). From this material we analyzed 16 samples covering six of the eight lithological units encountered in this Hole (I, II, IV, V, VI, and VII). Unit V consist of sedimentary breccia; likely as a result of a rock slide deposit, whereas all other units are represented by basalt, either as varying volcanic pillow basalt or massive flows with geochemical and petrographic distinct characteristics. Rock alteration can be assigned to low temperature processes.

In Hole 1383C the sediment/basement interface was found at 38.3 mbsf and 50.3 meter of hard rock was recovered from the interval between 69.5 and 331.5 mbsf (recovery 19%). The 17 samples investigated from this Hole are glassy to fine-grained basalts with variable content of phenocrysts, which divides them into three major lithological units (I, II, and III).

### DNA extraction

Small pieces of sample material was pulverized in a flame sterilized steel mortar and ~0.5 g (0.49–0.91 g) from each sample was subjected to genomic DNA extraction using the FastPrep soil DNA isolation kit (MP Biomedicals) following the manufacturer's instruction with two modifications. First, we used a special bead coating, similar to the G2 DNA/RNA enhancer (Amplicon A/S, Odense, Denmark, available from June 2016) that increases yield, by reducing DNA binding to the beads (Baælum and Jacobsen, 2010; Bælum et al., 2013; Hjelmso et al., 2014). Next, 200 μg of sterile filtered polyadenylic acid (PolyA; Sigma) was added to each lysis mixture prior to bead beating, to avoid DNA binding to the sample matrix (Hugenholtz et al., 1998). Bead beating was performed using the MP-Biomedical FastPrep®-24 for 45 s (speed setting 6). DNA was finally eluted into 75 μl PCR-grade double-distilled water (ddH_2_O), and preserved at −80°C until further analysis. In order to assess potential contamination introduced from the extraction kit, two blank extractions were included using the same batch of chemical reagents as for the samples.

### Quantitative PCR

Bacterial and Archaeal 16S rRNA genes were quantified individually using quantitative real time PCR applying the StepOne Real Time PCR system (Applied Biosystems). All samples and standards were run in triplicates using SYBR Green Hot Start master mixture (Qiagen) and with the standards, primers, and thermal conditions described in details elsewhere (Jørgensen et al., 2013). In short, a dilution series (10–10^6^ target copies) containing *Escherichia coli* PCR amplified full-length 16S rRNA genes and a linearized archaeal fosmid (54d9) was used as bacterial and archaeal standards, respectively. Bacterial SSU rRNA genes were targeted with the primers bac341F (5′-CCTACGGGWGGCWGCA) and 518R (5′-ATTACCGCGGCTGCTGG). For archaeal SSU rRNA gene amplification the primers Un515F (5′-CAGCMGCCGCGGTAA) and Arc908R (5′-CCCGCCAATTCCTTTAAGTT) were used. All *R*^2^ were >0.95 and the amplification efficiency between 90 and 104%.

### Ion torrent SSU rRNA amplicon library preparation and sequencing

All DNA extracts were PCR amplified in duplicates with the SSU rRNA gene specific primers 519f (5′-CAGCMGCCGCGGTAA) and 805r (5′-GACTACHVGGGTATCTAATCC) in order to generate an amplicon library for subsequent sequencing using the Ion Torrent PGM Personal Genome Machine (PGM) platform technology (Life Technologies). We used a two-step amplification approach as described by Berry et al. (2011), to minimize bias introduced by the long adaptor sequence. The first-round PCR was carried out in duplicate for each sample, to minimize PCR drifting, and each reaction (20 μl) contained 10 μl 2x HotStarTaq® master mixture (Qiagen), 0.2 μl of each primer (100 μM stock), 2 μl template and ddH_2_O. The PCR program was initiated with a hot start activation step for 15 min at 95°C followed by an optimized number of PCR cycles (36–37) of 95°C for 30 s, 56°C for 30 s, and 72°C for 30 s. The duplicate PCR products were pooled and purified using QIAquick PCR purification kit (Qiagen). In the second-round PCR attaching the Multiplex Identifiers (MIDs), seven cycles were run, where each reaction (25 μl) contained 12.5 μl 2x HotStarTaq® master mixture (Qiagen), 0.2 μl 806r-B-Key (100 μM stock), and 2 μl 519f MID primer (10 μM stock), with 5 μl of purified PCR products from first-round amplification as the template, according to the Ion Torrent protocol. The PCR amplicons were purified using AMPure XP bead Purification Kit (Agencourt), following manufactures protocol, before all samples were pooled in equimolar concentrations (26 pmol). We note, that due to the PCR and the subsequent equimolar pooling, the number of reads do not reflect the original concentration of DNA, which based on gel band intensity after PCR was much higher in the samples than in the blank extractions. Raw reads generated in this study were deposited at the NCBI Sequence Read Archive under the project number SRP070121.

### OTU filtering, clustering, and taxonomic assignment

Sequence reads obtained from the Ion Torrent sequencing were cropped at 220 bp and quality filtered with a 0.5 quality cut-off, chimera checked, and Operational Taxonomic Units (OTUs) clustered (97% similarity) using UPARSE/USEARCH (Edgar, 2013). The resulting OTUs were taxonomically assigned using the CREST software, with a lowest common ancestor algorithm implementing the SilvaMod reference database (Lanzen et al., 2012). A fasta sequence file of the represented OTUs can be found in the Supplementary Material (Data sheet S2).

### Contamination assessment of sequence pool

Contamination issues have previously hampered progress in deep marine research. In an attempt to avoid such obstacles this study applies several measures to prevent and assess the degree of potential contamination, as outlined in the sample handling and collection section above. Further, as a broad reaching contamination control for drilling protocols we extracted and sequenced DNA from the drill mud and a recovered microsphere bag (exposed to bottom seawater, drill fluid, and mud). The purpose of this control is to address potential inadvertent contamination of samples introduced during standard IODP drilling protocols. The results allow estimating the ratio of the inferred natural community that is likely to arise from contamination. Any OTU present in the control and in the native sample material was removed from the dataset. We note that true overlap between communities in the control and native samples may exist, which would lead to culling of legitimate sequences. Additionally, in order to assess potential contamination in the 16S rRNA gene amplicon preparation procedure two blank extractions (no sample material) were subjected to the same amplification protocol as the samples. Amplified DNA from these blanks may represent contamination originating from DNA extraction kit and/or PCR mix reagents (Champlot et al., 2010; Lusk, 2014; Salter et al., 2014). Therefore, any OTU found in both the extraction blanks and the native sample material was removed prior to any further downstream analysis, with the exception of OTUs that was found to be more than 50 times as abundant in the basalts than in the above-described controls. These were retained in the dataset, due to the plausible scenario of cross-contamination from controls to samples. This approach is similar to that described by Lee et al. (2015) with modified increased stringency addressing the assumed lower biomass in our sample set. However, in order to evaluate the impact of this stringent filtering, ordination, and clustering were likewise performed on the full dataset prior to the above described filtering.

### Ordination and hierarchical clustering analysis

The relative abundances of individual OTUs in each sample were clustered based on unconstrained Bray-Curtis, Jaccard, and Dice dissimilarity index using the software PAST version 3.08 (Hammer et al., 2001), before and after removal of potential contaminant reads. The basalt-hosted microbial communities were compared to those in the overlying sediments (Hole 1383E), using non-metric dimensional scaling (NMDS) applying Bray-Curtis dissimilarity. The data from the sediment samples was generated following identical protocols (e.g., the same primers, extraction kit, PCR mix), sequencing platform and downstream analysis, thereby enabling a valid comparison. The concentrations of major and trace elements from rocks provided by the IODP data report (Expedition 336 Scientists, 2012a,b) originating from the same core sections, but separated from the samples used for microbiology (between 20 and 70 cm distance), were used in cluster analysis using Bray-Curtis and Jaccard.

## Results

### 16S rRNA gene abundance

The total abundance of 16S rRNA gene copy numbers (Archaea plus Bacteria) estimated by qPCR varies between 0.3 and 8.3 × 10^4^ copies per gram in Hole 1382A and 0.6–3.9 × 10^4^ copies per gram in 1383C (Table 2), with the majority of all samples (80%) falling within the range of 1.9–5.8 × 10^4^ copies. Three samples from the sedimentary breccia between 162.8 and 163.8 mbsf in Hole 1382A (8R-2F, 8R-3G, and 8R-4D) had notably lower numbers (0.3–0.6 × 10^4^ copies per gram) than the remaining samples from this site. Based on our quantification the 16S rRNA genes are predominantly of bacterial origin, comprising between 92.6–100% and 91–100% in Hole 1382A and 1383C, respectively (Table 2). Assuming an average copy number per genome of 4.2 for Bacteria and 1 for Archaea (Stoddard et al., 2015), these copy numbers suggests cell abundances ranging ~0.1–2 × 10^4^ per gram of sample material (average 0.71 × 10^4^).

**Table 2:** **General molecular characteristic of the samples investigated**.

**Sample ID**	**Depth (mbsf)**	**Unit**	**16S rRNA copies (× 10^4^)**	**% Bacteria**		**Reads after filtering**	**OTU #**	**Shared with sediment**	
				**qPCR**	**Amplicon**			**OTUs**	**Reads of total %**
**U1382A**									
2R_1C	110	I	5.7	98	100	8,598	371	198	72
3R_2B	115	I	8.3	100	100	12,966	174	114	81
3R_3A	117	I	2.7	100	99	16,574	96	66	80
3R_4B	117	I	2.4	100	100	11,349	224	133	81
4R_1B	123	II	4.8	96	97	15,330	215	128	78
5R_1B	133	II	3.9	92	99	19,517	279	168	83
6R_1A	142	II	2.1	100	100	20,437	175	101	69
7R_2B	153	IV	3.8	100	100	10,968	193	127	85
8R_1A	161	V	1.8	100	100	23,251	42	23	80
8R_1B	161	V	4.1	95	100	12,495	261	142	80
8R_2F	162	V	0.3	100	100	19,858	16	10	99
8R_3G	163	V	0.5	100	100	19,145	25	18	22
8R_4D	163	V	0.6	100	86	25,028	27	20	31
9R_1C	172	V	4.2	92	99	16,873	314	185	82
10R_3D	183	VI	5.8	97	99	18,080	283	148	79
12R_1A	199	VII	3.0	92	98	19,506	273	153	75
**U1383C**									
2R_2E	72	I	2.8	99	100	19,163	319	165	63
3R_1B	77	I	3.1	98	100	18,240	141	75	75
4R_1B	87	I	1.9	98	97	19,144	124	72	72
5R_1BI	97	I	3.1	91	96	15,164	177	124	77
5R_1BII	97	I	2.6	99	99	19,553	330	168	75
6R_1A	105	I	2.2	100	100	23,627	243	146	71
10R_1A	144	II	2.1	90	100	25,841	158	99	86
10R_1D	145	II	2.9	98	100	17,447	326	161	83
11R_1C	154	II	1.7	99	100	24,686	204	116	87
19R_1B	212	III	3.0	98	100	19,107	264	130	84
19R_1A	212	III	3.3	99	100	18,946	126	81	88
20R_1A	219	III	2.9	100	100	3916	148	90	81
24R_1B	257	III	1.9	100	100	12,775	162	99	86
24R_1A	256	III	3.2	100	100	20,900	213	121	86
27R_1A	285	III	3.9	99	100	9,811	162	113	87
29R_1A	300	III	0.6	100	98	11,218	69	48	82
30R_1A	304	III	3.3	100	100	22,903	229	117	88

*Including gene copy numbers of total prokaryotic 16S rRNA gene copies per gram of sample material. Percent of the total community related to the domain Bacteria reported both from the qPCR and the amplicon library. The total number of reads after filtering. Number of OTUs in each sample (97% similarity). The number of OTUs and the % of total read that is shared with the samples in the above sediment from North Pond (1383E)*.

### Sequence reads, filtering, and OTU clustering

The total number of sequence reads per sample after filtering and potential contaminants removal (OTUs present in the four controls) varied between 8598 and 25,841 with an average of 17,649 (Table 2). A total of 1,804 OTUs (>97% sequence similarity) were found across all samples, of which 1,643 OTUs (91% of total OTUs) were assigned to the bacterial domain, 44 OTUs (2.5% of total OTUs) to the archaeal domain, while 60 OTUs (3.4% of total OTUs) were Eukaryotic. The remaining 57 OTUs were classified as “no hits” which means that the sequence is < 80% similar to any in the database (Data sheet S1). The number of OTUs in the individual samples varied between 16 and 371 (Table 2). Blank extractions contained a combined total of 221 OTUs of which 142 were removed according to the criteria outlined in the Materials and Methods Section. An additional 50 OTUs were removed from the original data, as they were present in either the drill mud and/or on the recovered microsphere bag (12 and 46 OTUs, respectively).

### Microbial community composition

**Figure 2:**
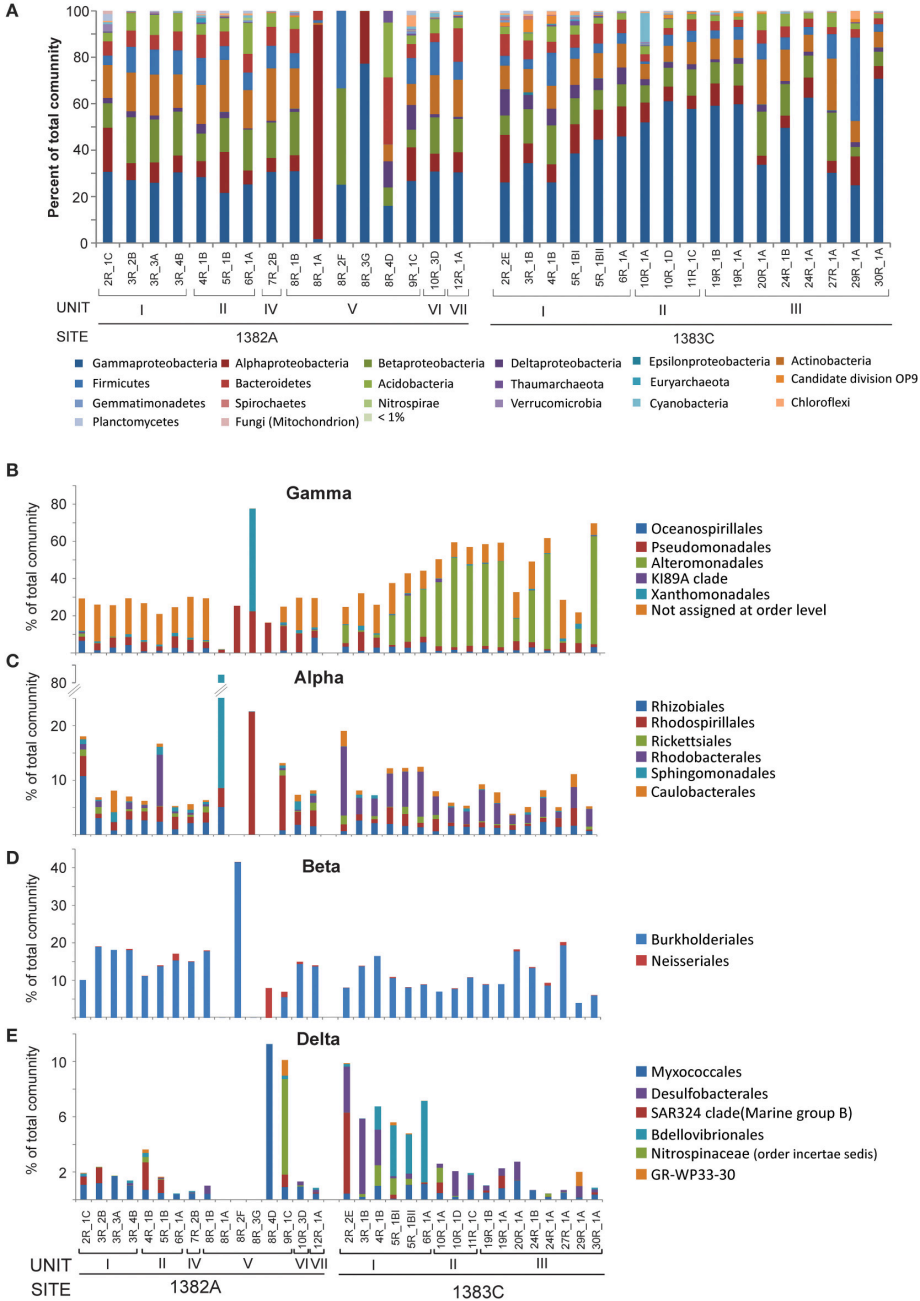
**Relative abundance of taxonomic groups. (A)** Class level abundances of *Proteobacteria* and phylum level for all other groups comprising more than 1% of total community in one or more samples. Abundances for the taxonomic orders representing more than 1% of total community of **(B)** *Gammaproteobacteria*, **(C)** *Alphaproteobacteria*, **(D)** *Betaproteobacteria*, and **(E)** *Deltaproteobacteria*.

As the microbial communities are spread across 41 different phyla, 73 classes, 155 orders, and 218 families, it is far beyond the scope of this work to address the community composition in all samples in detail. Therefore, only the results of the most abundant groups are listed here. However, a full list of OTUs present and their taxonomic assignments can be found in the Supplementary Material (Data sheet S1). In general the communities are relatively homogenous on higher taxonomic level across all samples, however in the sedimentary breccia the diversity of microorganisms and number of OTUs are extraordinarily low, especially in the middle section of that lithological unit (Figure 2 and Table 2), in many ways causing these samples to deviate substantially from the rest.

All samples are dominated by *Proteobacteria* (35–99% of the total communities) of which the class of *Gammaproteobacteria* is the most abundant, followed by *Alphaproteobacteria, Deltaproteobacteria*, and *Betaproteobacteria*, respectively (Figure 2A and Data sheet S1). Only very low abundances, if any, were assigned to the class of *Epsilonproteobacteria* and *Zetaproteobacteria*. Analysing the different classes of *Proteobacteria* with higher taxonomic resolution show that many (average 60%) of the *Gammaproteobacteria* could not be assigned below class level (Figure 2B). The majority of these were represented by OTU5, which showed high similarity (100%) to *Pseudomonas* when performing NCBI blast search. The limited taxonomic resolution of OTU5 in our analysis is likely due to the high stringency used by the lowest common ancestor algorithm applied to assign taxonomy. Most of the reminding *Gammaproteobacteria* in Hole 1382A belonged to the two orders *Oceanospirillales*, mainly affiliated with the SAR86 clade and *Pseudmonadales*, (largely divided between the families *Moraxellaceae* and *Pseudomonadaceae*). In contrast, the most abundant Gammaproteobacterial order from Hole 1383C is *Alteromonadales*, whereof most can only be assigned to family level (*Alteromonadaceae*) and to a lesser extent *Marinobacter* (< 1% of total community). Although, abundance-variation between samples is present within the *Alphaproteobacteria*, as for all taxonomic groups, *Rhizobiales, Rhodobaceriales*, and *Rhodospirilialles* were in general the most abundant orders (Figure 2C). At both sites, *Burkholderiales* was by far the most prominent member of the *Betaproteobacteria* at the order level, with the genus *Variovorax* accounting for approximately half of this group and the family *Oxalobacteraceae* representing the other half (Figure 2D and Data sheet S1). The overall abundance of *Deltaproteobacteria* was relatively low and in Hole 1382A it was dominated primarily by *Myxococcales* and the *SAR324* clade whereas *Bdellovibrionales* and *Desulfobacterales* were the most abundant orders in Hole 1383C (Figure 2E). In addition to the mentioned members of the *Proteobacteria* the following phyla were found in relatively high abundances; *Actinobacteria, Firmicutes, Bacteroidetes*, and *Acidobacteria* along with a number of less abundant groups, but still representing more than 1% of all reads in one or more samples, such as *Planctomycetes* and *Chloroflexi* (see Figure 2A).

The archaeal community constitute only a minor fraction of the entire community (max. 5%, avg. 0.6% of all reads) and is represented by five phyla; *Ancient Archaeal Group* (AAG), *Crenarchaeota, Euryarchaeota, Thaumarchaeota*, and the newly proposed *Lokiarchaeota* (Spang et al., 2015; Figure 2A and Data sheet S1). Of these, Thaumarchaeal *Marine Group I* is by far the most abundant.

A comparison between the rank abundance of the 576 OTUs shared between the basalt and the overlying sediment was performed using the average abundances of the OTUs across all basalt and across all sediment samples, excluding the sedimentary breccia from Hole 1384A (**Figure 5**). The shared OTUs represented between 63 and 86% (average 80%) of all reads in the basalt and 44–71% (average 58%) in the sediments, but show a markedly different rank abundance distribution (Table 2 and **Figure 5**).

### Ordination and clustering analysis

**Figure 3:**
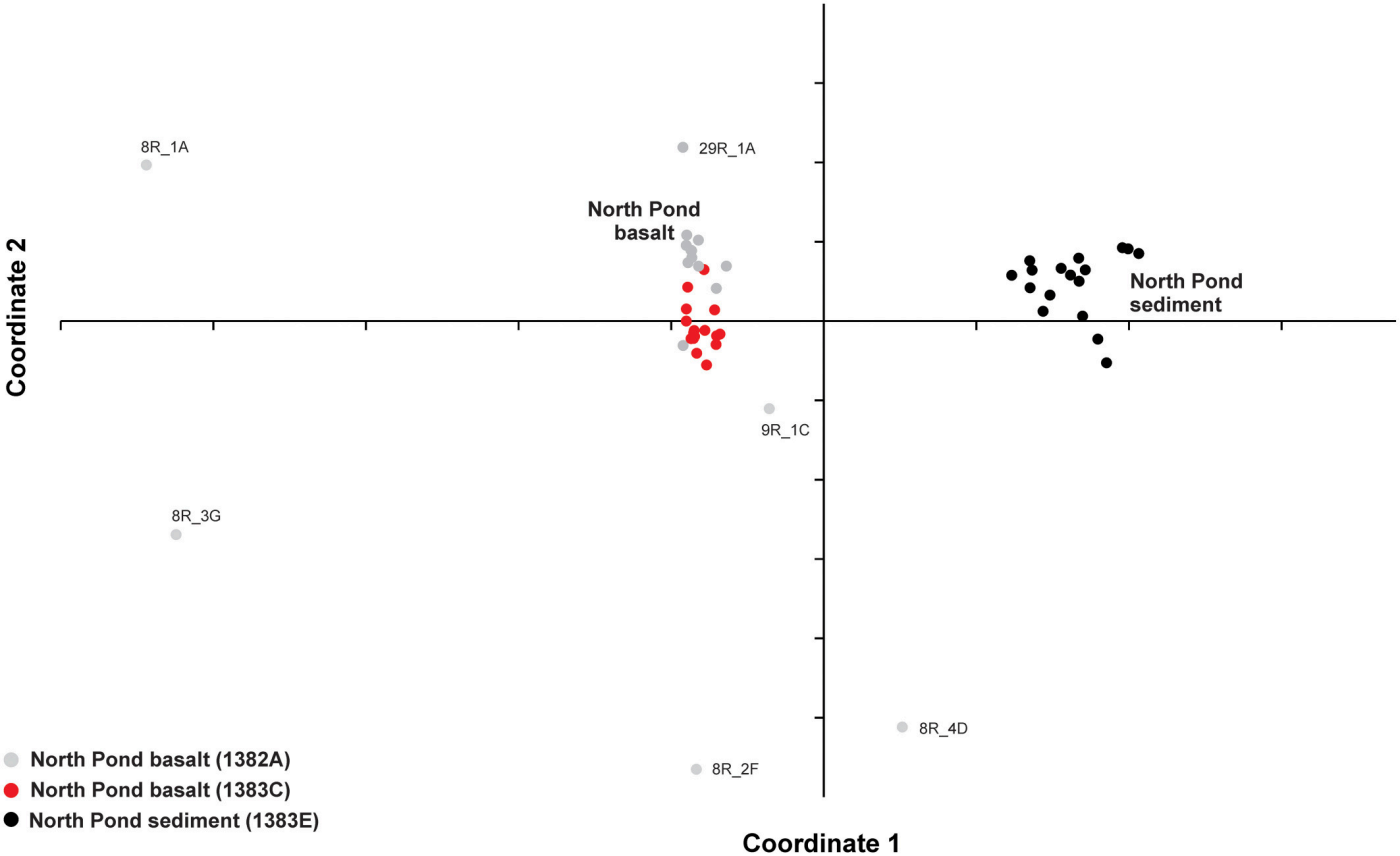
**Comparison between community structures**. Relative abundance of OTUs in each sample was used to compare the variation between the basalt-hosted communities and the sediment-hosted communities by means of non-metric multidimensional scaling (NMDS). Samples are color coded gray: Hole 1382A, red: 1383C, black: North Pond sediment from Hole 1383E.

The variation in the microbial community structure (relative abundance of OTUs) found in the basaltic samples beneath North Pond was compared to the composition in a number of sedimentary horizons in Hole 1383E directly overlying the basaltic crust by means of NMDS. The sedimentary community was investigated at 17 different depths, spanning from the top of the sediment to a few meters above the sediment basement interface and clearly shows a separation from those observed in the underlying upper crust (Figure 3). The microbial composition in the sedimentary breccia (lithological unit V) in Hole 1382A are markedly different from the rest of the samples from this sites and also from the basaltic rocks from Hole 1383C, causing these samples to cluster alone, with the exception of sample 8R_1B (the only basalt sample within the breccia unit). An NMDS analysis was also carried out on the full dataset, before removal of any of the OTUs found in the four controls, as described in the Materials and Methods Section. This result shows the same pattern, as with the “cleaned” dataset presented in Figure 3, and verifies that all control samples are markedly different from any of the indigenous samples (Figure S1).

**Figure 4:**
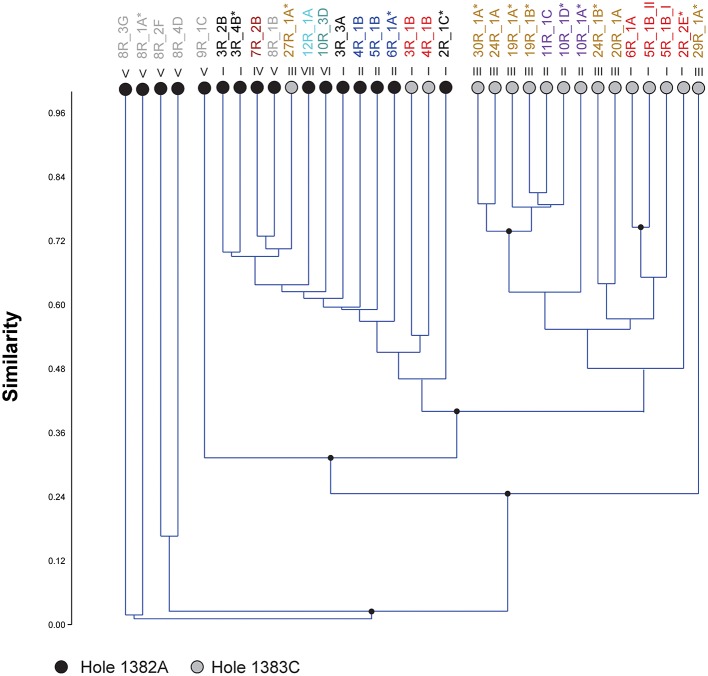
**Microbial community clustering**. Relative abundance of OTUs in each sample was used to cluster the different communities within the crustal samples from North Pond by means of Bray-Curtis distance calculations. Circles at the end of each branch indicates sampling site; gray: Hole 1383C, black; Hole 1382A. Roman numerals above the branches indicate the lithological unit, also indicated by colors in the sample name above. Asterisk indicates presence of microspheres in the wash fluids after last washing. Numbers above sample name indicates clusters supported by high bootstrap values (> 90) and are identical to numbers used in Table 2.

In order to investigate any link between community composition and lithology a hierarchical clustering analysis based on the relative abundance of OTUs was executed. The results show several minor (group 1, 2, 3, 6) and two major (group 4 and 5) clusters separated by high bootstrap value (Figure 4). The major clusters largely distinguish the two sites from one another. However, three samples from Hole 1383C cluster within 1382A. The bootstrap values are generally low and clustering according to lithology or depth cannot be inferred. The clustering using the full dataset, before removal of potential contaminant reads in general shows the same clustering pattern, however a higher degree of mixing between the two sites is observed (Figure S2). The results from clustering based on Jaccard and Dice dissimilarity indexes, showed no clear difference in clustering pattern as compared to Bray-Curtis (data not shown). Using the geochemical data (major and trace elements) from the same core sections as those used for microbiology, in a hierarchical clustering, shows only very small variation in composition. No clear clustering pattern between sites or lithology could be observed and most branching points were unsupported (Figure S3).

### Contamination control

The presence of fluorescent microspheres in the sterile seawater used to wash the basalt rock surface was investigated on board the ship. After three washing rounds, microspheres were detected in 36% of the samples (Figure 4) and more frequently observed in Hole 1383C than 1382A.

Drill mud and the microsphere bag yielded a total of ~47,500 high-quality reads comprising a number of different bacterial taxa (Data sheet S1). After removal of extraction blanks, 12 OTUs were obtained from the drill mud and 46 from the microsphere bag, most of these were affiliated with *Streptococcus*, a group that are often associated with human pathogens. A larger number of OTUs were obtained from the microsphere plastic bag, many of which were affiliated with different SAR clades. However, also here a number of reads were associated with bacterial groups often associated with humans (e.g., *Streptococcus* and *Dermabacter*; Data sheet S1). Standard drilling protocols inevitably introduce contaminants to drilling components and we propose this as an explanation for the detection of human associated taxa.

Extraction blanks were represented by ~16,000 reads whereof the vast majority could be assigned to the following three taxonomic groups: *Ralstonia, Enterobacteriaceae*, and *Methylobacterium*. On average 38% of all reads were removed during the cleaning procedure (total of 158 OTUs) highlighting the importance of performing operation controls and analysing blank extractions, especially when working with low biomass sample material (Data sheet S1).

## Discussion

### Microbial abundances in subsurface basaltic crust

Based on our qPCR results of prokaryotic 16S rRNA gene abundances, we estimate that the samples contain ~10^4^ cells per gram of rock sample, with *Bacteria* outnumbering *Archaea* in all samples (Table 2). However, as with all cell estimates based on a primer-based approach it is prone to bias and the numbers should be evaluated with this in mind. It is difficult to compare our estimates with earlier reports since, to the best of our knowledge, only one previous study has been conducted in which direct cell abundances were estimated from native cold subsurface basalt (Fisk et al., 2003). Based on amino acid concentration they suggest ~10^5^ cells per gram sample. A recent study estimated cell numbers in the basaltic fluids from both investigated sites, to be between 1.4 and 2.2 × 10^4^ per ml fluid, based on direct cell counts (Meyer et al., 2016). Considering the average basaltic porosity of 4% and assuming a density of 3, implies that our quantification is not merely representing cells in the fluid, but that the majority must be attached to the rock surfaces.

Due to the restricted number of sites in our study we have restrained ourselves from the tantalizing prospect of extrapolating the cell abundance to global biomass. However, we note that a previous estimate based on thermodynamic and bioenergetic models was suggested to translate into ~10^7^–10^9^ cells per gram rock (Santelli et al., 2008). In other words, 3–5 orders of magnitudes off our estimations. In support of a lower cell abundance is the relatively low oxygen consumption (< 1 nmol O_2_ cm^−3^ rock d^−1^) estimated beneath North Pond (Orcutt et al., 2013). Another interesting observation related to the cell numbers is the relative consistency across all samples, which suggest that the cells are limited by a common vital anabolic or catabolic resource.

### Microbial community composition in subsurface basaltic crust

Based on the 16S rRNA amplicon libraries the microbial communities in the subsurface basalt in North Pond are all dominated by Bacteria, in general leaving the archaeal domain represented by < 1%. Although, our qPCR estimations vary slightly from this, both analyses confirm the bacterial dominance (Table 2). By far the most abundant phylum was *Proteobacteria*, with *Gamma-, Beta*-, and *Alphaproteobacteria* constituting the majority within this phylum. In addition *Actinobacteria, Firmicutes, Bacteroidetes*, and *Acidobacteria* were all represented by relatively high abundances. To the best of our knowledge, only one previous published study has successfully amplified DNA from native subsurface material and determined the community composition (Lysnes et al., 2004). By means of DGGE that study reported sequences related to *Gammaproteobacteria, Actinobacteria, Bacteroidetes, Chloroflexi*, and *Firmicutes*, of which *Gammaproteobacteria* was the most abundant. A more recent study compiled taxonomic data from a number of studies regarding surface-exposed basalts and identified a set of commonly found abundant microbial groups, including *Gamma-, Alpha*-, and *Deltaproteobacteria*, as well as *Actinobacteria, Bacteroidetes, Acidobacteria, Planctomycetes, Gemmatimonadetes*, and *Nitrospirae* (Lee et al., 2015). Finally, the microbial community composition in the fluids underneath North Pond has revealed a similar dominance of *Proteobacteria*, also with *Gammaproteobacteria* being most abundant, then followed by *Alpha*-, *Epsilon*-, *Beta*-, and *Deltaproteobacteria* (Meyer et al., 2016). Further, relative high abundances of *Actinobacteria, Bacteroidetes, planctomycetes, Gemmatimonadetes*, and *Chloroflexi* were observed. Based on this it is tempting to suggest that a basalt-hosted taxonomic core group exists, including surface, subsurface fluids, and hard rocks. However, it is important to stress that the similarity of microbial groups in all cases is based on high taxonomic rank and future in-depth phylogenetic comparison, founded on full-length 16S rRNA sequences, are needed to resolve this issue.

**Figure 5:**
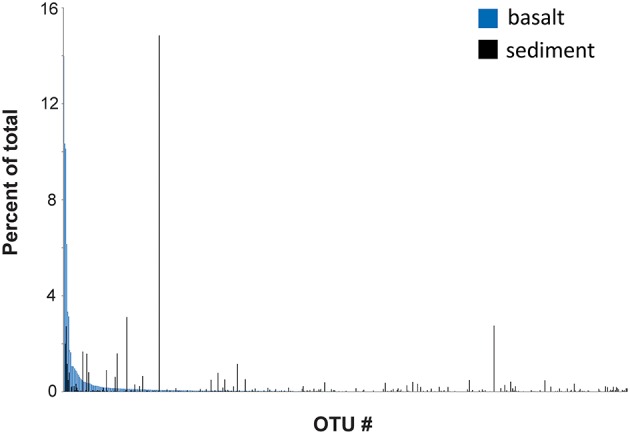
**OTU rank abundance**. Comparing the rank abundance of OTUs shared between the basalt-hosted community (Holes 1382A and 1383C) and the sediment-hosted (1383E). The abundance of each OTU across all basalt-hosted samples and across all sediment samples was compiled and the average abundance used (excluding the sedimentary breccia in Hole 1382A).

The origin of the subsurface oceanic crustal community is an open question and different scenarios have been debated, one of them being that dispersion may occur via the above sediment column (Huber et al., 2006; Schrenk et al., 2010). When we compared the relative community composition in the basaltic rocks with that in overlying sediment striking differences were observed (Figure 3). However, this is by no means indicative of community isolation between the crust and sediment. On the contrary, a high degree of overlap was revealed from the OTUs distribution pattern. Of the 1,802 OTUs found across all 33 crustal samples, 576 OTUs were shared with the sedimentary habitat above, comprising as much as 63–86% (average 80%) of all reads in the basaltic samples. However, the relative distributions of the shared OTUs in the two environments are markedly different, which might reflect environment-specific taxon recruitment based on availability of electron donors and acceptors (Figure 5). Based on these results the two components of the deep biosphere do indeed share many of their microbial inhabitants. Although, the dynamics and mechanisms of taxon dispersal between the crustal and sedimentary subseafloor regimes is beyond the scope of this study, we note that a recent study support the dispersal of sedimentary bacteria via the ocean (Walsh et al., 2016).

Regarding the heterogeneity of crust-associated microbial communities beneath North Pond, we note that although the communities from the two different sites to a large degree cluster together, (Figure 3), there are also clear differences (Figure 4). Two major clusters, supported by high bootstrap values (> 90) were found, largely separating the two investigated sites from one another. Notable differences are the presence of a few highly abundant OTUs affiliating with an uncultured *Alteromonas* lineage (*Gammaproteobacteria*) in Hole 1383C and a much higher occurrence of *Rhodobacterales* (*Betaproteobacteria*) in Hole 1383C than in Hole 1382A. The significance of this is not known, but it is possible that the physico-chemical nature, and associated redox coupling, of advective crustal fluids, drive community differentiation.

### Potential metabolic traits and dominant groups

The vast majority of lineages reported here do not group within taxonomic clades with known metabolism, and therefore their potential role in the ecosystem is unresolved. However, a number of less abundant groups with relatively constrained metabolic potential are present, thereby allowing us to assign their function with some degree of certainty. For example, as putative sulfate reducers in other environments a number of different genera were observed, including, *Desulfotomaculum, Desulfurispora, Desulfosporosinus, Desulfobaca, Desulfobulbus, Desulforomonas, Desulfovibrio*, and *Desulfobacula*, suggesting that at least the potential for active sulfate reduction is present. The classical sulfur oxidizers (mainly within *Epsilonproteobacteria*), on the other hand, were only sporadically observed. This observation is worth mentioning in the context of the relatively high abundances of sulfur oxidizers found in the fluids by Meyer et al. (2016) and in the overlying sediments, suggesting different functions between the free-living and the surface-attached communities.

Iron and hydrogen (beside sulfur) has been proposed to be important electron donors in this type of habitat (Bach and Edwards, 2003; Edwards et al., 2005). However, known metal reducers such as, *Marinobacter, Shewanella, Geobacter*, and *Ferruginibacter* made up only a minor fraction of the entire community (*Marinobacter* up to 0.3%). Hydrogen utilization is another widespread trait that is difficult to pinpoint based on taxonomy alone, hence we could only assign this to *Hydrogenophilus, Hydrogenophaga*, and *Paracoccus*, all represented in the dataset, but in low abundances.

Finally, we observe a number of groups with the ability to transform nitrogen compounds. This includes members of the putative ammonium-oxidizing archaeal Marine Group I (< 5% of total community) and nitrifiers (*Nitrospira*), which was detected in discrete samples up to 0.5%. Despite, the relative low abundances, their presence suggest an active nitrogen cycle, which is in congruence with the low concentration of organic carbon measured in this environment (Orcutt et al., 2015; Sakata et al., 2015).

### Contamination control

A great concern related to investigations of subsurface crustal material (and the deeply buried biosphere in general) has been the challenge of overcoming issues related to contamination constrains (Lever et al., 2006; Santelli et al., 2010). In order to delineate potential sources of contamination (acquired during drilling or subsequent sample processing) we analyzed two sample processing controls (extraction blanks) and two drilling operation controls (drill mud and a recovered empty microsphere bag). Designation of potential contaminants facilitates the tracking of their source in addition to their downstream removal from the bioinformatic pipeline as described before. As more reads were removed from the native samples due to their overlap with extraction blanks rather than with the drill mud and microsphere bag, we conclude that more contamination was introduced during DNA extraction and amplification than from the actual drilling procedure. However, we acknowledge that contamination could have originated from drilling or downstream procedures not accounted for by our contamination controls.

Despite, the observed contamination, several lines of evidence suggest that the final community structure is not corrupted: (i) the bifurcation of community structure of the two crustal sites, (ii) drastic differences between the composition and structure of sedimentary and crustal samples, despite equally low biomass, (iii) congruence between our results and taxonomic identity of enriched organisms from independent studies at this site, including *Pseudomonas, Burkholderia, Bacillus, Salinibacterium, Sphingomonas, Moraxella*, and *Methylobacterium* (Hirayama et al., 2015), (iv) agreement with core-taxa hitherto identified in basalt hosted environments (Lysnes et al., 2004; Lee et al., 2015) and in the fluids under North Pond (Meyer et al., 2016).

In sum, the data shows that contamination in low abundance habitats is a concern, and we encourage that both extraction and operation controls are performed. If such measures are taken the influence of contamination in data analysis seems manageable.

We also note that the presence of microspheres, a general measure of contamination deployed during IODP drilling operations, does not seem to be reflected in the magnitude of contamination in the 16S rRNA gene libraries (measured as number of reads removed in the filtering due to drill mud contamination).

## Conclusions

Our study gives some of the first insights into the microbial inventory of the subsurface oceanic crust in a young cool ridge flank system and show a community dominated by *Proteobacteria* (*Gammaproteobacteria, Alphaproteobacteria*, and *Betaproteobacteria*), followed by *Actinobacteria, Firmicutes, Bacteroidetes*, and *Acidobacteria*. In general, the same phyla are present in high abundances on seafloor-exposed basalts and in the crustal fluids, suggesting the possibility for a common basalt-hosted microbial biome. However, more data is needed to establish if a core group also exist at deeper taxonomic levels. Within the crustal communities we find microbial representatives that are likely to be involved in iron, sulfur, hydrogen, and nitrogen cycling but all in relative low abundances. However, the activity levels might be considerably higher than what the relative abundance implies.

Our estimated cell abundances are on average 0.7 × 10^4^ cells per gram of igneous rock, several orders of magnitude lower than what has been found on seafloor-exposed basalt (e.g., Einen et al., 2008; Santelli et al., 2008). The abundance is relatively consistent across all samples and we therefor speculate that the available energy is equal across the different samples and/or cell numbers are limited by a common nutrient factor.

Further, the variations in community structure between the samples do, to a large extent, separate the two investigated sites (1382A and 1383C) into two major clusters with no apparent link to differences in lithology. Comparing the basalt-hosted community structure to the overlying sediment using the relative distribution of individual OTUs clearly separates the two environments. However, ~1/3 of all OTUs in the basalt, representing an average of 80% of all sequences, were also present in the overlying sediment, indicating coupling between the two compartments of the deep biosphere. This observation is interesting with respect to the origin of the microorganisms inhabiting the crustal aquifer, however, deciphering the direction of cell dispersal, the source and the nature and underlying mechanisms of such links needs further investigation.

## Author contributions

SJ designed the research; SJ and RZ performed the research; SJ and RZ analyzed the data; and SJ and RZ wrote the manuscript.

### Conflict of interest statement

The authors declare that the research was conducted in the absence of any commercial or financial relationships that could be construed as a potential conflict of interest.
