# Supplementary material for: Microbial Inventory of Deeply Buried Oceanic Crust from a Young Ridge Flank
Source: Front Microbiol. 2016 May 27;7:820. doi: 10.3389/fmicb.2016.00820 (PMC4882963; doi:10.3389/fmicb.2016.00820)

Figure S1. Comparison between community structures. Using the full OTU dataset without removal of contaminants, otherwise similar to Figure 3. Relative abundance of OTUs in each sample was used to compare the variation between the basalt-hosted communities and the sediment-hosted communities by means of non-metric multidimensional scaling (NMDS). Samples with red label are the four controls. The five basalt outliers are labeled with sample name in blue, the rest in-circled and labeled native basalt North Pond. Sediment-hosted communities (Hole 1383C) are in-circled and labeled Sediments North Pond.

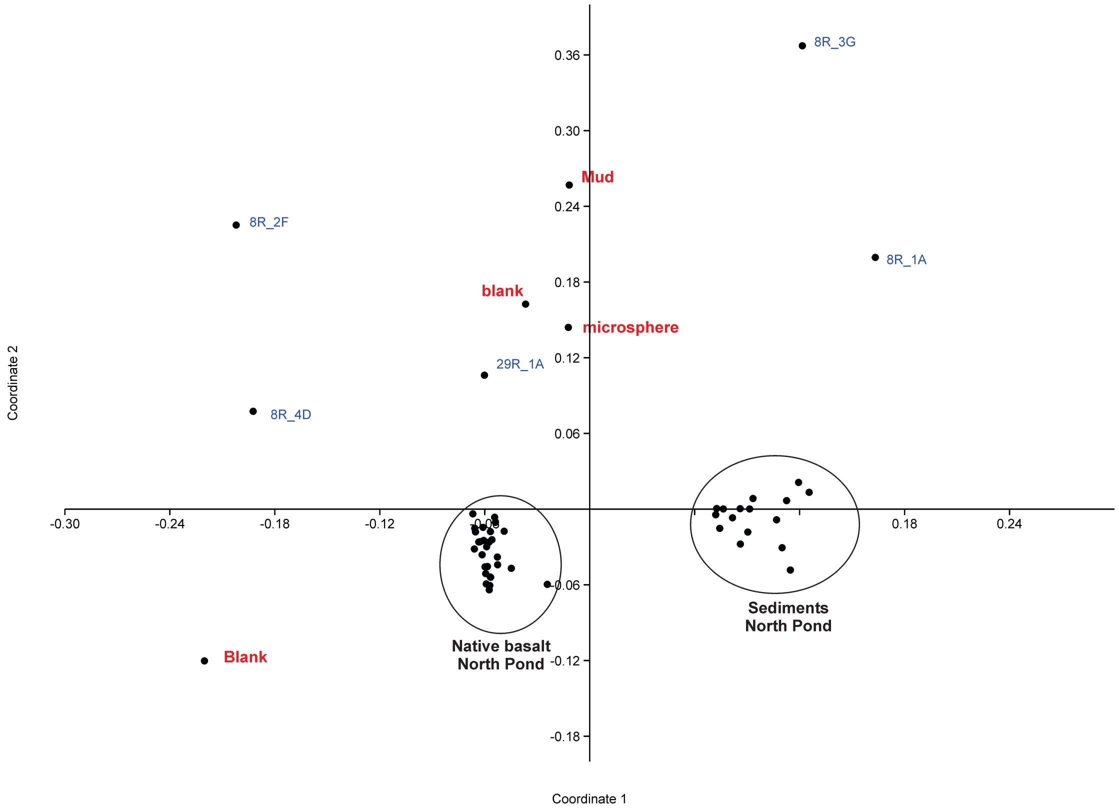

Supplement: Supplementary file 3 [file Image1.PDF]
