# Supplementary material for: Microbial Inventory of Deeply Buried Oceanic Crust from a Young Ridge Flank
Source: Front Microbiol. 2016 May 27;7:820. doi: 10.3389/fmicb.2016.00820 (PMC4882963; doi:10.3389/fmicb.2016.00820)

Figure S2. Microbial community clustering. Using the full dataset without removal of contaminants, other wise similar to Figure 4. Relative abundance of OTUs in each sample was used to cluster the different communities within the crustal samples from North Pond and the four controls by means of Bray-Curtis distances calculations. Sample name is given at the end of each branch.

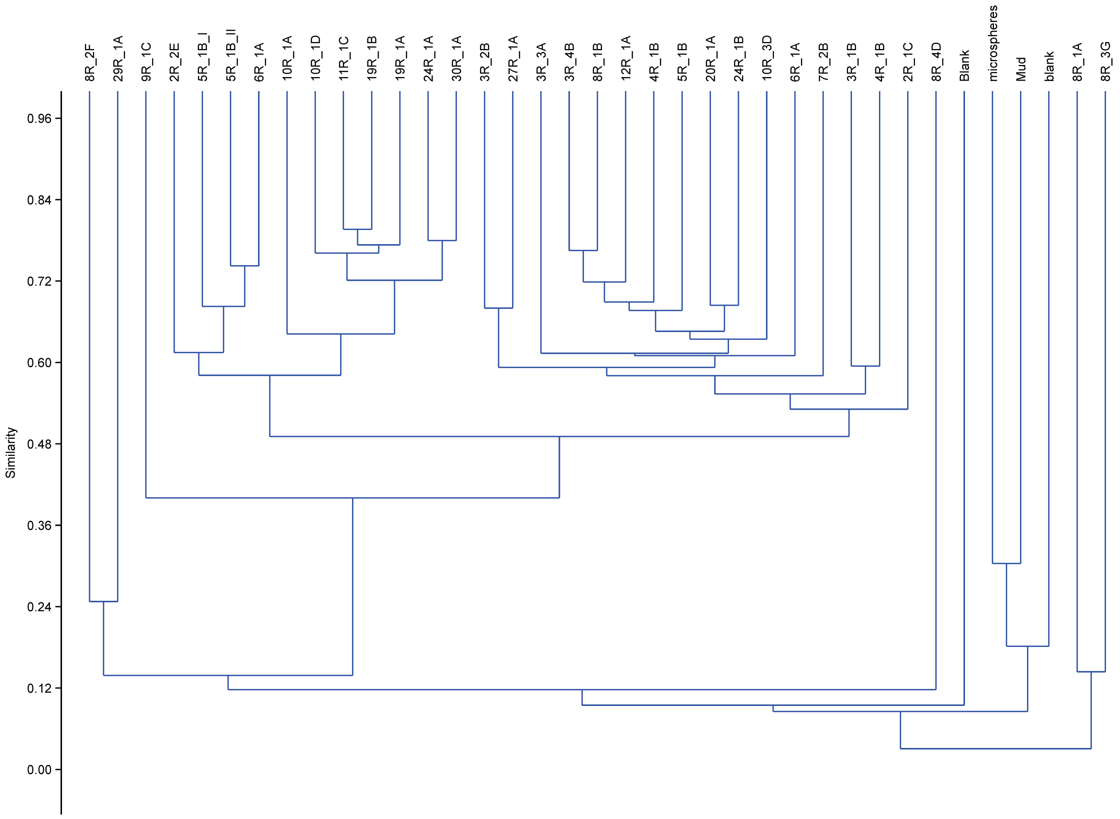

Supplement: Supplementary file 4 [file Image2.PDF]
