# Supplementary material for: Microbial Inventory of Deeply Buried Oceanic Crust from a Young Ridge Flank
Source: Front Microbiol. 2016 May 27;7:820. doi: 10.3389/fmicb.2016.00820 (PMC4882963; doi:10.3389/fmicb.2016.00820)

Figure S3. Geochemical clustering. Using the concentration of major and trace elements by means of Bray-Curtis distances calculations. Sample name is given at the end of each branch. Gray circles indicate samples from 1383C and black circles 1384A. Roman numerals indicate the lithological unit.

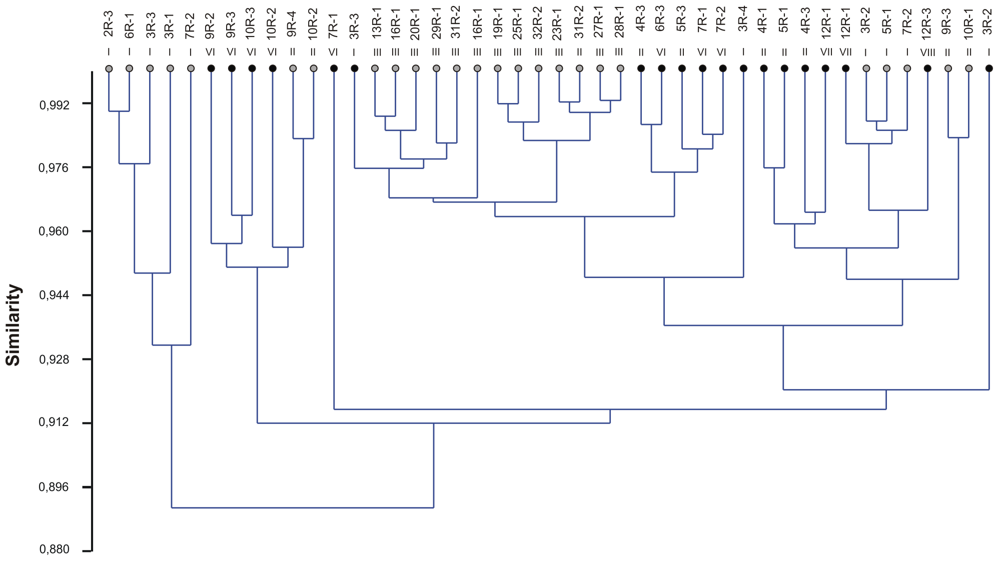

Supplement: Supplementary file 5 [file Image3.PDF]
